# Elevated atmospheric carbon dioxide and plant immunity to fungal pathogens: do the risks outweigh the benefits?

**DOI:** 10.1042/BCJ20230152

**Published:** 2023-11-17

**Authors:** Freya Smith, Estrella Luna

**Affiliations:** Birmingham Institute of Forest Research, School of Biosciences, University of Birmingham, Edgbaston Campus, Birmingham B15 2TT, U.K.

**Keywords:** climate change, CO_2_, crops, forest trees, plant defence, resistance mechanisms

## Abstract

Anthropogenic emissions have caused atmospheric carbon dioxide (CO_2_) concentrations to double since the industrial revolution. Although this could benefit plant growth from the ‘CO_2_ fertilisation’ effect, recent studies report conflicting impacts of elevated CO_2_ (eCO_2_) on plant–pathogen interactions. Fungal pathogens are the leading cause of plant disease. Since climate change has been shown to affect the distribution and virulence of these pathogens, it is important to understand how their plant hosts may also respond. This review assesses existing reports of positive, negative, and neutral effects of eCO_2_ on plant immune responses to fungal pathogen infection. The interaction between eCO_2_ and immunity appears specific to individual pathosystems, dependent on environmental context and driven by the interactions between plant defence mechanisms, suggesting no universal effect can be predicted for the future. This research is vital for assessing how plants may become more at risk under climate change and could help to guide biotechnological efforts to enhance resistance in vulnerable species. Despite the importance of understanding the effects of eCO_2_ on plant immunity for protecting global food security, biodiversity, and forests in a changing climate, many plant–pathogen interactions are yet to be investigated. In addition, further research into the effects of eCO_2_ in combination with other environmental factors associated with climate change is needed. In this review, we highlight the risks of eCO_2_ to plants and point to the research required to address current unknowns.

## Background: rising atmospheric carbon dioxide

Unprecedented greenhouse gas (GHG) emissions have fuelled global climate change in recent decades [1]. Gases including carbon dioxide (CO_2_), methane (CH_4_), and nitric oxide (NO) contribute to the greenhouse effect by trapping infrared radiation in the atmosphere, generating accelerated warming and climate change. CO_2_ accounts for 76% of GHG emissions [2] making it the most prevalent in the atmosphere. Anthropogenic CO_2_ emissions from fossil fuel combustion and industry are the primary source of CO_2_ and have increased atmospheric CO_2_ concentrations since the industrial revolution, rising from 280 parts per million (ppm) in 1750 to 416 ppm in 2022 [3,4]. In high emission scenarios, the Intergovernmental Panel on Climate Change (IPCC) predicts doubling of annual anthropogenic CO_2_ emissions from 40 gigatonnes in 2015 to 80 gigatonnes by 2050 [5], thus resulting in an atmospheric concentration in the figure of 550 ppm. In addition, degradation of carbon sinks like forests and peatlands increase CO_2_ concentrations by releasing stored carbon [6]. With rising emissions, carbon sink preservation is essential to attenuate climate change.

Climatic effects of CO_2_ emissions have been reported in the air, oceans, ice, and on land, varying from increased temperatures, raised sea levels, reduced ice sheets, and longer growing seasons, respectively [7]. Rising surface temperatures increase severe weather events like droughts, storms, and heatwaves. Additionally, glacial and ice sheet reductions have accelerated rising sea levels and flooding [7]. These current environmental conditions under climate change can alter the length of growth seasons and create abiotically stressful habitats for plants and animals, whilst also affecting biotic stressors like fecundity, virulence and spatial ranges of pathogens and pests [8]. Therefore, attempts to reduce GHG emissions are vital to mitigate negative repercussions on biota.

Importantly, higher atmospheric CO_2_ has direct impacts on plants, generally enhancing photosynthesis and growth [9]. However, whether future increased CO_2_ concentrations will benefit plants overall remains unclear. Recent studies have focused on exploring whether enhanced plant resources could impact immunity against pests and pathogens. Most of those studies have found that plant pathogen exposure to eCO_2_ resulted in an impact to pathogenicity and plant resistance phenotypes to viral, bacterial, fungal, or oomycete infections. However, the direction of the effect on resistance phenotypes, whether positive or negative, was highly diverse. From all these biotic threats, the impact of eCO_2_ against fungal pathogens seems to be the most controversial. Considering the impact of fungal pathogens on plant health and survival, this paper reviews current research into the effects of eCO_2_ on plant pathogenesis and resistance phenotypes against these pathogens. In addition, we discuss our findings in the context of different factors of global change, evaluate the threats and opportunities CO_2_-enrichment provides plant systems, and aim to answer the question of whether the potential benefits in plant growth and development outweigh the risks to plant immunity and disease incidence.

## The threat of fungal pathogens

Fungal pathogens cause the majority of plant diseases and are a major issue in agriculture [10]. For instance, severe diseases in cereal crops can cause serious yield reductions [11], endangering food security and economic stability. A specific example is the fungal pathogen *Fusarium oxysporum,* which infects many non-cereal plant species, and in banana plants could have the potential to fully destroy this crop when in monoculture cultivation [12]. Additionally, major forest tree losses have been attributed to fungal infections. For instance, Dutch Elm Disease, caused by the fungus *Ophiostoma novo-ulmi,* is responsible for the death of millions of elm trees across the world and despite many scientific studies, it still represents a huge problem [13]. More recently, the fungal pathogen *Austropuccinia psidii* has reportedly pushed rainforest Myrtaceae trees towards extinction [14]. Unfortunately, the consequences associated with fungal infections in forest systems go beyond the threats to biodiversity. Forests play a crucial role for carbon capture and storage; therefore, a reduction in forest cover accelerates climate change by no longer providing storage capacity. For example, ash trees are threatened by ash dieback disease caused by the fungal pathogen *Hymenoscyphus fraxineus.* A healthy mature ash tree may capture up to 22 kg of atmospheric CO_2_ each year [15]; however, ash dieback kills the trees once infected, thus preventing carbon storage. Additionally, infected ash trees are either left to decompose or burnt to prevent pathogen spreading, which again releases stored CO_2_ back into the atmosphere. Plant fungal diseases therefore pose a significant risk towards the implementation of strategies to mitigate climate change.

In recent decades, several analyses have reported a poleward spread of fungal pathogens and an overall increase in fungal disease incidence [16,17]. This has been attributed to changes in temperature, precipitation, and humidity associated with climate change, permitting increased survival and success of fungal pathogens in areas outside their typical ranges. For instance, the first outbreak of wheat blast disease in Bangladesh coincided with significantly higher temperatures and humidity levels which the causal fungal pathogen *Magnaporthe oryzae* requires for development [18]. Similarly, emergence of an economically important fungal pathogen of wheat and maize, *Fusarium verticillioides,* has increased at higher latitudes in Europe as a result of warmer temperatures benefiting the pathogen and facilitating the growth of its host plants in new areas [19]. Increased vulnerability of trees due to environmental stresses including drought, flooding, and high temperatures, as well as milder, wetter conditions favouring pathogen spread, also likely contributed to the devastating recent expansion of ash dieback disease throughout Europe [20]. Fungal pathogens are therefore a current and increasingly prevalent threat to agriculture, forestry, and biodiversity around the world.

## The arms race between plants and fungal pathogens

Plants and fungal pathogens are in constant evolutionary fight. Pathogenic fungi infect plants through spores which adhere to plant surfaces and germinate. Penetration peg formation facilitates entrance into the plant cell. This occurs using cell-wall degrading enzymes, turgor pressure for direct piercing and in some cases (e.g. rust fungi) by stomatal openings [21]. Subsequent infection differs based on pathogen lifestyle. Biotrophic fungal pathogens require living plant tissues for survival, therefore their infection and formation of feeding structures do not affect the viability of host cells [22]. In contrast, necrotrophic pathogens benefit from host cell death. Necrotrophs produce toxic and cell-degrading compounds which kill plant cells for the pathogen to then consume [23]. Hemibiotrophs initially infect hosts biotrophically, permitting the survival of plant cells, then later transcriptionally reprogramme to a necrotrophic lifestyle and cause necrosis.

At the other end of the arms race, plants have physical and chemical defences against fungal infections. As a physical barrier against infection, plants have thick cuticle layers composed of cutin and wax [24]. However, pathogens can overcome this pre-contact defence; for example*,* the fungal pathogen *F. oxysporum* can secrete cutinases to degrade the host plant's cuticle layer and infect cells [24]. As a result, plants also possess a double-layered innate immune system against pathogens. The first layer of defence, PAMP-triggered immunity (PTI), occurs when plant pathogen recognition receptors (PRRs) recognise conserved pathogen-associated molecular patterns (PAMPs) like chitin in fungal cell walls [25]. This recognition triggers a signalling cascade leading to transcription of defence-related genes. One of the fastest plant defence responses is the production of reactive oxygen species (ROS), such as hydrogen peroxide (H_2_O_2_), which can have antibiotic, cell-wall strengthening or loosening, and secondary messenger abilities [26]. As part of the immune response, PAMP recognition also triggers stomatal closure to block pathogen entry [26] and up-regulates production of secondary metabolites such as phytoalexins which, along with constitutively produced phytoanticipins, are toxic to fungal pathogens [27]. Other secondary metabolites synthesised upon pathogen recognition include defence-related phenolic compounds. Total soluble phenolics (TSPs) are thought to provide a barrier to infection progression through antifungal and cell-wall strengthening properties [28]. Infection progression is also restricted by increased biosynthesis and deposition of lignin and callose in cell walls at pathogen invasion sites [29] and the production of antimicrobial compounds such as tannins [30]. The PTI response can also involve production of antifungal proteases like chitinase [26].

The second layer of innate immunity is required against effector proteins which pathogens evolved to suppress PTI. For instance, the fungus *Cladosporium fulvum* secretes apoplastic effector Avr2 to inhibit plant proteases [31]. Plant NLRs (nucleotide-binding leucine-rich repeat proteins) recognise effectors and activate effector-triggered immunity (ETI). This often produces the hypersensitive response of controlled cell death and activates the synthesis of phytohormones salicylic acid (SA), jasmonic acid (JA), and ethylene (ET) [32]. The SA-signalling pathway induces systemic acquired resistance (SAR) which provides whole-plant resistance to a range of pathogens. SAR is often accompanied by the production of PR proteins which contribute to fungal pathogen resistance. SA is generally thought to be involved in coordinating plant defence responses against biotrophic pathogens, whereas JA and ET are thought to coordinate defence against necrotrophs [33]. The role of phytohormone signalling in plant defence responses is complex as the regulatory hormones often interact and overlap [22]. For instance, SA and JA generally maintain an antagonistic effect, which has been characterised in many plant species; however, the hormones can also act synergistically in plant defence [34]. Plants can also sensitise their defence mechanisms through priming of defence [35,36]. Priming is a component of the plant immune system where a plant once exposed to a pathogen will, upon subsequent infection, produce quicker and stronger defence responses [37]. Therefore, whereas fungal pathogens possess different infection strategies and can switch between different lifestyles, plants are equipped with prodigious defence mechanisms which allow them to fight pathogenic infections.

Elevated atmospheric eCO_2_ has been shown to have many effects on plants and their fungal pathogens. In C3 plants limited by CO_2_ availability, eCO_2_ promotes photosynthesis over photorespiration, generally increasing plant biomass and development through the ‘CO_2_ fertilisation’ effect [38]. Growth under eCO_2_ has also been shown to affect plant water use efficiency, carbohydrate and leaf nitrogen concentrations, stomatal behaviour through CO_2_ signalling, and respiratory rates [38]. However, the direction and extent of these changes in plants can be species-specific and dependent on other environmental factors. For instance, the effects of eCO_2_ on biomass and leaf nitrogen differed between four different grass species and was dependent on soil fertilisation levels [39]. Moreover, plant internal molar fractions of CO_2_ under eCO_2_ concentrations have been proven to be difficult to determine and this is mostly due to the actual effect of the stomatal density and closure in different plant species and parts of the leaves [40]. In addition to plant growth and development, eCO_2_ can also modify plant metabolism and impact defence signalling pathways [38]; thus, under eCO_2_, the arms race between plants and their fungal pathogens becomes even more complex.

Research has suggested that higher atmospheric CO_2_ concentrations have directly and indirectly contributed to the increased incidence of fungal disease reported in recent years [16,17]. High CO_2_ has been shown to directly affect pathogen aggressiveness and spore production. For instance, fecundity of the ascomycete *Colletotrichum gloeosporioides* increased under eCO_2_ (700 ppm), resulting in greater disease severity in shrubby stylo (*Stylosanthes scabra*) [41]. Indirectly, eCO_2_ can increase the incidence of fungal disease through physiological changes to host plants. Greater plant photosynthetic rates under eCO_2_, especially in C3 plants, may provide more host tissue biomass for fungal invasion. This can also increase canopy size and leaf longevity to create a preferable microclimate for spore trapping and proliferation [42,43]. Along with increased population size under eCO_2_, a favourable microclimate could accelerate pathogen evolution. However, increased plant growth can also be accompanied by lower disease incidence [44]. Greater carbon acquisition under eCO_2_ can shift plants’ carbon-nitrogen balance, typically resulting in lower leaf nitrogen levels which differentially affect disease severity depending on the pathogen's specific nutritional limitations [45]. Nevertheless, the effect of eCO_2_ on plant tissue quality has also been shown to be species-specific [39]. Moreover, changes in stomatal behaviour through CO_2_ sensing under eCO_2_ can also affect fungal pathogens that utilise stomatal pores for infection [46].

Despite sophisticated plant defence mechanisms, rapid pathogen evolutionary rates can result in plant exposure to novel effectors [47], increasing susceptibility to infection and weakening resistance from fungicides. Therefore, the potential acceleration of pathogen evolution under eCO_2_ [48] highlights the importance of understanding factors influencing plant defence in order to develop alternative ways of enhancing resistance. Due to frequently observed benefits of eCO_2_ on photosynthesis, water-use, and nutrient-use efficiency, one might hypothesise that the same benefits occur for plant immunity [49]. Many studies have been conducted into the impact of eCO_2_ on host–pathogen interactions upon fungal attack with limited consensus. Future CO_2_ concentrations may benefit plants in some ways; however, complex immune responses can be expected, along with varying outcomes on pathogenicity. Studies in recent years report highly controversial phenotypes on the effect of eCO_2_ in plant immunity. In this article, we have reviewed the literature for enhanced, attenuated, or unchanged immunity under eCO_2_ to shed light into the plant defence mechanisms that could play a role in these multifaceted plant–pathogen interactions.

## Positive effects of eCO_2_ on plant immunity

**Figure 1.**
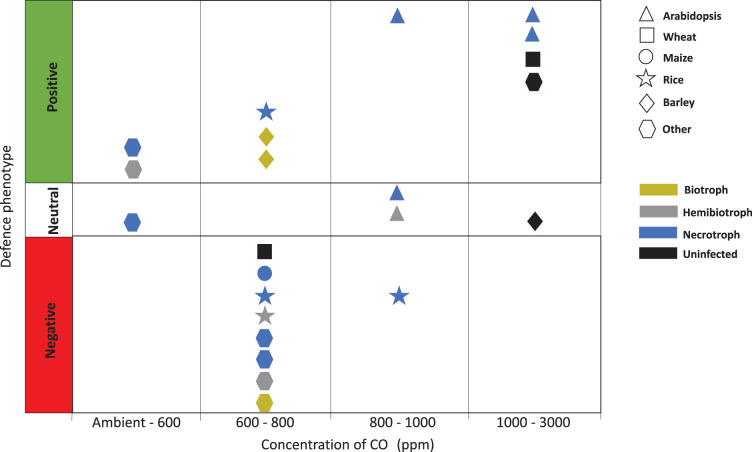
Schematic to summarise papers reporting enhanced (positive), attenuated (negative), or unchanged (neutral) immune phenotypes in important plant species under elevated CO_2_ (parts per million, ppm). Each point represents a study's conclusion on the overall effect of eCO_2_ on a plant species’ immunity. Mustard, grey, and blue coloured points represent plants inoculated with a biotrophic, hemibiotrophic or necrotrophic fungal pathogen, respectively. Black points are studies where plant immunity was analysed in the absence of infection. Studies of Arabidopsis (triangles), wheat (squares), maize (circles), rice (stars), and barley (diamonds) were included for comparison. ‘Other’ species (hexagons) include studies on chickpea, tea, bean, cucurbits, tomato, mustard, and red maple.

Some studies have found a positive effect of eCO_2_ on plant immunity and resistance to fungal pathogens (Table 1). Many of these studies have focused their efforts on unravelling the role of plant defence hormones in the activation of defence responses. It is generally accepted that SA-signalling enhances plant resistance to biotrophic fungal pathogens and suppresses necrotrophic resistance by the JA-pathway [50]. Plants often accumulate greater SA concentrations under eCO_2_ [51]; consequently, this could promote resistance to biotrophs and increase necrotrophic infections. However, several studies indicate that plant defence responses to eCO_2_ are more complex. Williams et al. [52] reported that eCO_2_ enhanced resistance of *Arabidopsis thaliana* to necrotrophic *Botrytis cinerea*, accompanied by both JA- and SA-pathway up-regulation (Table 1). In agreement, Mhamdi and Noctor [34] found greater SA accumulation in Arabidopsis, wheat (*Triticum aestivum*), and bean (*Phaseolus vulgaris*), and a combined up-regulation of JA- and SA-dependent genes in Arabidopsis. The authors suggested that the combined up-regulation of these generally antagonistic phytohormones may have been permitted by intracellular oxidative stress caused by growth under eCO_2_. Their more detailed analysis found increased levels of resistance genes and antifungal anthocyanins in Arabidopsis grown at eCO_2_ compared with ambient CO_2_ (aCO_2_). This experiment went further to suggest that eCO_2_ causes priming of defence, rather than full activation of the immune response, since some SA accumulation occurred when eCO_2_ was tested alone. However, one problem with these results is that leaf chemistry was measured after only 4 weeks at eCO_2_. Consequently, this study cannot fully represent future plant responses to long-term eCO_2_. This is partially true for some of the studies showing contrasting phenotypes since CO_2_ concentrations used (Table 1; Figure 1) were unrepresentative of realistic future levels [53]. Interestingly, although also reporting that eCO_2_ enhanced Arabidopsis resistance to *B. cinerea*, Zhou et al. [54] oppose Mhamdi and Noctor [34] by arguing that marker genes for JA-dependent defence were up-regulated while SA-related genes were down-regulated. This highlights the complexity of immunity even within the same plant–pathogen species interaction, suggesting that different growth conditions between studies contribute to plant phenotypes. Unlike Zhou et al. [54], Williams et al. [52] considered how enhanced developmental rates at eCO_2_ can influence defence signalling [55] and ensured that eCO_2_-grown plants were compared at the same developmental stage. This makes their argument of combined JA and SA up-regulation more convincing.

**Table 1:** Plant species that showed enhanced resistance to fungal pathogens under eCO_2_

Plant species	Pathogen	Pathogen lifestyle	Concentration of eCO_2_ (ppm^1^)	Reference
Arabidopsis (*Arabidopsis thaliana*)	*Plectosphaerella cucumerina*	Necrotrophic	1200	[52]
Arabidopsis (*Arabidopsis thaliana*)	*Botrytis cinerea*	Necrotrophic	800	[54]
Arabidopsis (*Arabidopsis thaliana*)	*Botrytis cinerea*	Necrotrophic	3000	[34]
Common bean (*Phaseolus vulgaris*)	*Botrytis cinerea*	Necrotrophic	3000	[34]
Common wheat (*Triticum aestivum*)	*Botrytis cinerea*	Necrotrophic	3000	[34]
Rice (*Oryza sativa*)	*Cochliobolus miyabeanus*	Necrotrophic	700	[28]
Red maple (*Acer rubrum*)	*Phyllosticta minima*	Necrotrophic	560	[42]
Mustard (*Brassica juncea*)	*Alternaria brassicae*	Hemibiotrophic	550	[56]
Barley (*Hordeum vulgare*)	*Erysiphe graminis*	Biotrophic	700	[57]
Barley (*Hordeum vulgare*)	*Blumeria graminis*	Biotrophic	700	[58]

1 Indicates parts per million;

Common and species names of studied plants are listed, along with species and lifestyle of the invading fungal pathogen.

Studies at more realistic CO_2_ concentrations also report enhanced defence against necrotrophic fungal pathogens. In rice (*Oryza sativa*) [28] and red maple (*Acer rubrum*) [42]; eCO_2_ increased secondary metabolites, namely phenolic compounds and tannins, as well as lignin for structural support. The study into red maple is especially significant since, despite the widely understood importance of trees as carbon sinks [6], they are markedly underrepresented in the literature and the effects of eCO_2_ on immunity of many species remains unknown. Greater phenolic compound production also occurred in mustard (*Brassica juncea*) along with increased JA and resistance to *Alternaria brassicae* [56]*.* However, the methodological approaches of growing plants in open top chambers (OTCs) [28,56] and Free Air CO_2_ Enrichment (FACE) facilities [42] raises concerns over the results’ robustness. Although results from OTCs and FACE experiments may better represent natural plant responses to eCO_2_, they reduce control over other environmental factors. Possible interactions with herbivorous insects were not controlled or recorded in these experiments, nor was the average temperature, or humidity in [56] and [42]. These factors can influence defence responses [51,54]; therefore, these papers cannot undoubtably conclude enhanced immunity was due to eCO_2_ alone. Further research into the combined influence of these environmental factors is therefore necessary.

## Negative effects of eCO_2_ on plant immunity

Several rather puzzling scenarios were found in the literature, with many papers directly contradicting positive findings and reporting attenuated immunity to fungal pathogens under eCO_2_ (Table 2). For instance, while studies by Mhamdi and Noctor [34] and Zhou et al. [54] argued that eCO_2_ enhanced resistance to *B. cinerea* in Arabidopsis with increased JA (Table 1), Zhang et al. [50] found that in infected tomatoes (*Solanum lycopersicum*) eCO_2_ repressed JA signalling and favoured the SA pathway, reducing resistance against necrotrophs. They reported this was due to eCO_2_ increasing antagonistic SA and JA crosstalk through the *nonexpressor of pathogenesis-related genes 1* (NPR1) of the SA signalling pathway. This was supported by a later study in tomato, which found the same interaction [59]. The contrasting results between studies in Arabidopsis and tomato support the idea that species-specific genetic and developmental variation can cause different phenotypes within pathosystems [60]. Notably, different growth conditions were used between studies. Zhang et al. [50] grew plants at higher temperatures with shorter photoperiods compared with Mhamdi and Noctor [34]. Recently, elevated temperatures and longer photoperiods were suggested to suppress and enhance immunity, respectively [61,62]. This may have varied host susceptibility, but the photoperiod was shorter in the positive study by Zhou et al. [54] compared with Zhang et al. [50]. This suggests that plant immunity is influenced by the interplay of many factors which should be comprehensively investigated in future research. Zhang et al. [50] exposed plants to eCO_2_ for just four days compared with four weeks in the other two studies. Consequently, it cannot represent changes in immunity which may occur under sustained eCO_2_ in the future. Researchers should be cautious of their investigation's claims as prolonged exposure could have given different results.

**Table 2:** Plant species that showed reduced resistance to fungal pathogens under eCO_2_

Plant species	Pathogen	Pathogen lifestyle	Concentration of eCO_2_ (ppm^1^)	Reference
Tomato (*Solanum lycopersicum*)	*Botrytis cinerea*	Necrotrophic	800	[50]
Tomato (*Solanum lycopersicum*)	*Botrytis cinerea*	Necrotrophic	800	[59]
Rice (*Oryza sativa*)	*Rhizoctonia solani*	Necrotrophic	600–680	[69]
Rice (*Oryza sativa*)	*Magnaporthe oryzae*	Hemibiotrophic	600–800	[69]
Rice (*Oryza sativa*)	*Fusarium fujikuroi*	Necrotrophic	850	[70]
Common wheat (*Triticum aestivum*)	^2^	Biotrophic	700	[68]
Maize (*Zea mays*)	*Fusarium verticillioides*	Necrotrophic	800	[65]
Tea (*Camellia sisnensis*)	*Colletotrichum gloesporioides*	Hemibiotrophic	800	[63]
Cucurbits (*Cucurbita pepo, Lagenaria siceraria, Luffa cylendrica*, *Cucumis sativus, Momordica charantia*)	*Sphaerotheca fuliginea*	Biotrophic	600	[64]

1 Indicates parts per million;

2 This study did not infect plants with a fungal pathogen;

Common and species names of studied plants are listed, along with species and lifestyle of the invading fungal pathogen.

In tea (*Camellia sinensis*), eCO_2_ worsened hemibiotrophic *Colletotrichum gloeosporioides* infection as foliar caffeine concentrations, which increase JA biosynthesis, diminished [38]. This opposes positive effects on hemibiotrophic infection in mustard [56] (Table 1), suggesting that eCO_2_ can distinctly affect resistance against pathogens with the same lifestyle. The more detailed study by Mathur et al. [56] measured changes in SA, PR proteins, and phenolic compounds, as well as JA transcripts. Furthermore, mustard was grown at eCO_2_ for 8 weeks longer than tea and had more biological replicates, perhaps making their results more robust. Importantly, Li et al. [63] found that exogenous application of caffeine re-enhanced tea plant resistance against fungal infection. Applying plant defence compounds to induce defence is an emerging area of research. Greater exploration into this technique may allow the development of a novel and more environmentally safe alternative to synthetic fungicides to commercially enhance resistance and protect future food security in an elevated CO_2_ world.

In cucurbit species including pumpkin (*Cucurbita pepo*), eCO_2_ repressed plant immunity by decreasing foliar SA and phenolic compound concentrations [64]. In agreement, Vaughan et al. [65] found decreased JA, SA, and phytoalexin production in maize following *F. verticillioides* infection. As a plant that uses the C4 photosynthetic pathway, which can concentrate low levels of CO_2_ for more efficient photosynthesis, limited benefits of eCO_2_ may influence maize defence responses [66]. Despite their importance for food security [67], C4 crops such as sorghum, sugarcane, and maize are rarely investigated in eCO_2_-immunity experiments. Due to the fact C4 plants often show reactions under eCO_2_ distinct to C3 species [66], we believe that further investigation into how their immune responses may vary is necessary.

In wheat, contrary to positive immune responses reported by Mhamdi and Noctor [34], Kane et al. [68] found eCO_2_ attenuated immunity through repressing ET and JA signalling. Protein kinases involved in defence against *Puccinnia striiformis f. sp. tritici* also decreased; however, since this study failed to investigate the effects of eCO_2_ on immunity in plants inoculated with a fungal pathogen, the results may not be replicated in real-life scenarios with a present infection. It should also be noted that this study reported positive effects of eCO_2_ in wheat acclimated to cold temperatures (5°C), finding up-regulation of PR protein and SAR-related genes. Clearly, studies should account for interplay of multiple environmental parameters, including seasonal and geographical weather variations to better predict future plant responses.

Contradicting the positive effects of eCO_2_ on rice immunity reported by Dorneles et al. [28]. Kobayashi et al. [69] reported greater rice susceptibility to *Rhizoctonia solani* and *Magnaporthe oryzae* in a FACE experiment. These results were attributed to lower leaf silicon concentrations as eCO_2_ lessened transpiration. Silicon assists plant immunity through enhancing phenolic compounds, PR protein and phytoalexin production, regulating signalling, and forming physical barriers to infection [18]. Notably, increased susceptibility to *M. oryzae* was not consistently recorded in the experiment; one year of investigation showed no significant difference between plants at eCO_2_ and aCO_2_. This was suggested to be due to relatively longer photoperiods and lower humidity; however, changes in leaf silicon were insignificant, suggesting that untested defence mechanisms may have been affected. This highlights a gap for investigation into possibly interacting effects of light, humidity, and eCO_2_ on immunity. Despite their importance for investigating plant responses to eCO_2_ under natural conditions, the impact on plant immunity has been tested in only a few FACE experiments [42,69]. Opposing Kobayashi et al. [69], Hibberd et al. [57] reported eCO_2_ increased silicon and enhanced resistance of barley to a biotrophic fungal pathogen (Table 1). This again indicates distinct defence responses between plant–pathogen systems and may support the suggestion eCO_2_ favours resistance against biotrophs [51]. However, these studies are limited by not investigating changes in defensive phytohormone or secondary metabolite levels, basing their claims solely on silicon concentrations. In a more detailed study of rice, Matić et al. [70] found eCO_2_ down-regulated genes required for the PTI response, resulting in weakened defence against *Fusarium fujikuroi*.

## Neutral effects of eCO_2_ on plant immunity

Despite many papers concluding that eCO_2_ influences plant immunity against fungal pathogens, a few others found no significant effects (Table 3). Contradicting and therefore weakening their overall argument that eCO_2_ enhanced plant immunity against *B. cinerea*, Mhamdi and Noctor [34] found no effect in barley, showing contrasting responses between plant species. This further supports the idea of distinct phenotypes between pathosystems, which may explain why eCO_2_ enhanced barley resistance to a different pathogen in a previous study [57].

**Table 3:** Plant species that showed no significant change in resistance to fungal pathogens under eCO_2_

Plant species	Pathogen	Pathogen lifestyle	Concentration of eCO_2_ (ppm^1^)	Reference
Barley (*Hordeum vulgare*)	*Botrytis cinerea*	Necrotrophic	3000	[34]
Arabidopsis (*Arabidopsis thaliana*)	*Rhizoctonia solani*	Necrotrophic	800	[54]
Arabidopsis (*Arabidopsis thaliana*)	*Fusarium oxysporum f.sp. raphani*	Hemibiotrophic	800	[54]
Chickpea (*Cicer arietinum*)	*Fusarium oxysporum*	Necrotrophic	550	[72]

1 Indicates parts per million;

Common and species names of studied plants are listed, along with species and lifestyle of the invading fungal pathogen.

In Arabidopsis, eCO_2_ enhanced resistance to *B. cinerea* but had no significant effect on *Rhizoctonia solani* and *Fusarium oxysporum f.sp. raphani* infection [54]. This occurred despite up-regulation of JA-defence marker genes under eCO_2_ before infections were performed. One potential weakness of this paper is that changes in defence after infection were not quantified by altered host physiology, but instead by disease severity. Due to only measuring defence-related genes prior to infection, this study missed the opportunity to explain their arguments perhaps through finding that the JA increase under eCO_2_ was insufficient, not sustained, or influenced by other mechanisms upon infection. In opposition, immunity against *R. solani* was significantly weakened in rice [69]. This more detailed study provided evidence for reduced defence by recording silicon levels. Conflicting results again suggest distinct species phenotypes due to genetic and environmental variation. Unlike artificially inoculated Arabidopsis, *R. solani* naturally infected rice plants in Kobayashi et al. [69] perhaps making their results more valid to real-life plant–pathogen interactions. However, since inoculation methods can impact disease occurrence and severity [71], this may have influenced the results and makes direct comparison challenging.

Interestingly, in disagreement to all studies reporting a significant effect of eCO_2_, Bhatia et al. [72] found that chickpea (*Cicer arietinum*) only showed enhanced defence against *F. oxysporum* when ozone was also elevated (eO_3_). Without eO_3_, eCO_2_ caused no significant difference in PR protein β-1,3-glucanase or peroxidases involved in lignin and phytoalexin production. The relatively lower eCO_2_ concentration (550 ppm) used in this study, which is closer to predicted near-future levels [53], may explain the neutral effect of eCO_2_ and make the results more useful. This study's novel result may have also been influenced by using soil-inoculation which can be less effective at inducing disease symptoms than the foliar spore-sprays or droplets used in most other studies [73]. In contrast, another study found eCO_2_, eO_3_ and elevated temperatures individually enhanced immunity in barley against the biotrophic fungus *Blumeria graminis.* However in combination, these factors resulted in the same immunity observed under ambient conditions [58] (Table 1, Table 3). Together, these studies emphasise a need for more studies to investigate the combined effects of several environmental factors associated with climate change on plant immunity.

## eCO_2_ and its impacts on key defence mechanisms

**Figure 2.**
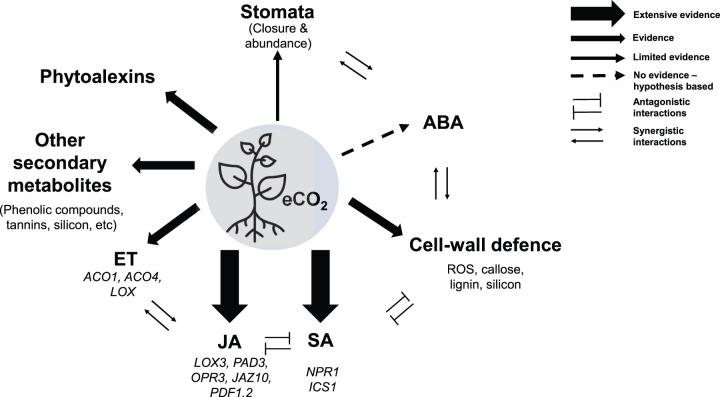
Schematic summary of the interactions between eCO_2_ and major defence signalling pathways, defence mechanisms of plant immunity and synergistic/antagonistic interactions between defence mechanisms. Arrows from the centre represent an interaction of eCO_2_, with the thickness of arrows correlating with the amount of evidence in the current literature. Dashed lines show speculative interactions with no current experimental evidence.

This review highlights the many impacts that eCO_2_ has on plant defence mechanisms against fungal pathogens (Figure 2). From our literature search, it is clear that eCO_2_ crucially affects SA and JA biosynthesis and signalling, as effects on these key defence hormones were reported in many different publications. Whereas the exact processes by which eCO_2_ alters these phytohormone signalling is unknown, there is strong evidence of different hormone synthesis and signalling genes being influenced by eCO_2_ (Figure 2). For instance, evidence of eCO_2_ altering the expression of key SA synthesis gene, *isochorismate synthase1* (ISC1), and JA synthesis genes *lipoxygenase3* (LOX3), *12-Oxophytodienoate reductase 3* (OPR3)*, jasmonate-zim-domain protein 10* (JAZ10), and *plant defensin 1.2* (PDF1.2) was reported in Arabidopsis [34]. Generally, eCO_2_ has been shown to enhance and repress SA- and JA-dependent mechanisms, respectively, which corresponds with the default direction of the well-studied antagonistic crosstalk between SA and JA. However, publications in Arabidopsis and mustard have reported an enhanced expression of JA-dependent genes and the down-regulation of SA-dependent genes [54,56], highlighting that eCO_2_ can also trigger antagonistic effects in the other direction. Interestingly, a different publication in Arabidopsis has shown that eCO_2_ activates both SA and JA-dependent defences at the same time, thus erasing the SA and JA antagonistic crosstalk [34]. Therefore, whereas it is evident that eCO_2_ impacts SA and JA, its effect in their defence crosstalk is extremely complex.

In addition to the already complicated effects of eCO_2_ in key defence hormones, secondary metabolites, cell-wall defences, stomatal responses and other phytohormones are also known to be impacted by eCO_2_ (Figure 2). For instance, in different plant pathosystems, eCO_2_ has been shown to both enhance and attenuate the production of phytoalexins, phenolic compounds, tannins, and other antifungal secondary metabolites, which contribute to plant defence [18,42].

Studies have also shown that eCO_2_ can impact concentrations of lignin [28], callose [74], and silicon [69], in addition to affecting ROS homeostasis [75], all of which impact the state of cell-wall fortification against biotic stress. However, investigations into the effects of eCO_2_ on these defences in plants facing fungal pathogen invasion are limited, with research into callose- and ROS-based cell-wall defences restricted to plant responses to insect [74] and parasitic weed infestations [75], respectively. Moreover, these cell-wall strengthening mechanisms interact further with hormonal signalling (Figure 2); crosstalk of SA and JA with callose deposition [76], lignin biosynthesis [77], ROS bursts [78], and redox signalling [34] has been reported.

eCO_2_ can also impact phytohormones besides SA and JA; however, they are less well studied (Figure 2). The effect of eCO_2_ on one of the most important defence-related phytohormones, ET, is highly controversial and studies into the interaction with fungal pathogen infections are absent. In non-infected wheat, Kane et al. [68] reported that eCO_2_ decreased the JA and ET synthesis transcripts of *1-aminocyclopropane-1-carboxylate oxydase* (ACO) and *lipoxygenase* (LOX), with similar results reported in soybean (*Glycine max*) infested with Japanese beetle (*Popillia japonica*) [79]. Conversely, eCO_2_ up-regulated the ACO genes ACO1 and ACO4 in non-infected tomato [80]. Moreover, understanding the effect of eCO_2_ on ET biosynthesis and plant defence is further complicated by the known synergistic and antagonistic crosstalk of ET with JA and SA, respectively (Figure 2).

Another contributing factor to the impact of eCO_2_ on plant immunity is its influence on stomatal behaviour. Again, conflicting results have been reported between papers and are influenced by interactions with other plant defence mechanisms (Figure 2). For instance, in uninfected Arabidopsis leaves, eCO_2_ led to lower stomatal density and conductance [81]; however, in Arabidopsis under fungal attack, eCO_2_ increased stomatal density, which may have facilitated easier colonisation of the fungus *Erysiphe cichoracearum* in newly developed leaves [48]. Lake and Wade (2009) hypothesised that interactions between eCO_2_ and the MAPK kinases (MAPKs) controlling Arabidopsis stomatal development may have disrupted normal stomatal behaviour in response to pathogen infection [48]. They also highlighted how synergistic crosstalk between MAPKs and abscisic acid (ABA) signalling, as well as complex influences of JA, SA, and ET, also play a role in determining the effect of eCO_2_ on stomatal behaviour.

ABA is one of the most important plant hormones involved in responses against abiotic and biotic stress. ABA has been proven to be a highly controversial plant hormone in terms of its role in defence. For instance, it has been described that the plant species, the concentration of ABA, and the pathogen lifestyle hugely impact whether ABA exerts a positive or negative result on resistance [82]. Remarkably, scientific evidence into the effect of eCO_2_ in ABA biosynthesis and signalling is lacking and the only reference of a role of eCO_2_ impacting ABA-dependent defences is based on speculations on its role on stomata closure and abundance [83] (Figure 2). Considering the central role of ABA in stomata closure and antagonistic and synergistic effects with other defence mechanisms (i.e. ET, SA, JA, cell-wall defence [84]), we are able to hypothesise that contrasting effects of eCO_2_ in immunity could be dependent on ABA. In turn, the lack of research on the role of eCO_2_ in ABA highlights the bigger gap in our understanding of the interactions between plant responses to abiotic and biotic stresses at the same time.

## Effects of CO_2_, a complex process

The primary literature contains conflicting arguments of how eCO_2_ affects plant immunity against fungal pathogens (Figure 1). Although a few studies found no change in plant defences, the majority support the hypothesis that eCO_2_ triggers changes in immunity. Biotrophic, necrotrophic, and hemibiotrophic pathogen infections were all associated with both enhanced and attenuated plant immunity under eCO_2_ (Figure 1), suggesting that pathogen lifestyle, plant species, and environmental factors all act together to determine immune responses. Species-specific responses are also suggested by the fact eCO_2_ had only positive or neutral effects on Arabidopsis infected by different pathogens (Figure 1). As described above, contrasting defence phenotypes may be due to the effects of eCO_2_ in plant developmental processes, which vary hugely between plant species. For instance, stomatal abundance, closure, and the internal molar fraction of CO_2_ under eCO_2_ has been shown to vary not only between plant species but also between different parts of the leaves [40]. Considering the relevance of stomatal behaviour as a first barrier of defence against certain fungal pathogens (i.e. rust fungi) but not all, these factors could perhaps contribute to explaining some of the contrasting immune phenotypes reported. In addition to stomatal behaviour, eCO_2_ has diverse effects on phytohormones, antimicrobial secondary metabolites, and cell-wall defences, all of which interact to influence plant immunity (Figure 2). Consideration of many plant defence mechanisms simultaneously is therefore necessary to understand specific plant responses to pathogen infection under eCO_2_.

This review also highlights a need for greater study into biotrophic and hemibiotrophic infections since research appears disproportionately focussed on necrotrophs (Figure 1). Many of the most devastating plant diseases are caused by biotrophs and hemibiotrophs. For instance, Septoria leaf blotch, one of the most important wheat diseases in Europe [85], is caused by the hemibiotrophic fungus *Zymoseptoria tritici,* however how this interaction may be affected by eCO_2_ remains unknown. Since pathogens with a biotrophic phase survive on living material, improved growth rates of plants under eCO_2_ may increase the success of these pathogens, making understanding the impact on plant defence especially important. Overall, the different effects of eCO_2_ on plant immunity and pathogen virulence, as well as the influence of factors such as temperature, humidity, and photoperiod [54,61] make predicting outcomes on defence difficult. Changes in these environmental parameters under climate change means future research should explore their individual and overlapping effects on immunity to provide more accurate predictions of potential trends and direct biotechnological interventions accordingly.

## Conclusion

The existing literature indicates that plant growth under eCO_2_ has varying effects on immunity against fungal pathogens, being largely influenced by species and interplay with environmental factors. Despite recent research, knowledge is still limited; numerous causal pathogens of major diseases and responses of many plant species, such as C4 crops necessary for food security and many tree species, remain untested. Current understanding is especially inadequate due to the specificity of interactions between different plants, pathogens, the combination of multiple abiotic factors under climate change, and the complex interactions among different defence mechanisms. Consequently, the risks of eCO_2_ to plant immunity outweigh potential benefits in plant growth since so much remains unknown. Although plant responses to fungal infection under future eCO_2_ cannot entirely be represented by artificial inoculation and experimental conditions, continuous and more comprehensive investigation could help anticipate future outcomes on the health and survival of different plants. This would benefit understanding of future threats to global food security, biodiversity, and forest systems and help to direct conservation efforts towards vulnerable species.

## Open Access

Open access for this article was enabled by the participation of University of Birmingham in an all-inclusive *Read & Publish* agreement with Portland Press and the Biochemical Society under a transformative agreement with JISC.

## Abbreviations

ABA: abscisic acid
aCO_2_: ambient CO_2_
ET: ethylene
FACE: free air CO_2_ enrichment
GHG: greenhouse gas
JA: jasmonic acid
MAPKs: MAPK kinases
NO: nitric oxide
OTCs: open top chambers
PTI: PAMP-triggered immunity
ROS: reactive oxygen species
SA: salicylic acid
SAR: systemic acquired resistance
